# Supplementary material for: Health promotion intervention to prevent risk factors of chronic diseases: Protocol for a cluster randomized controlled trial among adolescents in school settings of Chandigarh (India)
Source: PLoS One. 2022 Feb 17;17(2):e0263584. doi: 10.1371/journal.pone.0263584 (PMC8853575; doi:10.1371/journal.pone.0263584)
Supplement: S1 File — (DOCX) [file pone.0263584.s002.docx]

**Thesis Plan**

**Health Promotion Intervention Package for Prevention of Behavioral Risk Factors of Chronic Diseases: A Cluster Randomized Controlled Trial among Adolescents in School Settings**

Submitted in the partial fulfillment

for the degree of

DOCTOR OF PHILOSPHY


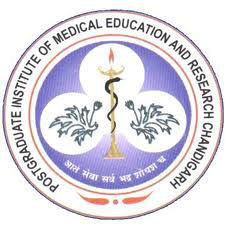


of the

Post Graduate Institute of Medical Education and Research, Chandigarh

Submitted by

Sandeep Kaur

School of Public Health

**Co- guide**

Dr. Rajesh Kumar

Ex Dean (Academic) & Ex- HoD, DCM & SPH, PGIMER, Chandigarh

**Guide**

Dr. Manmeet Kaur Professor (Health Promotion) Department of Community Medicine and School of Public Health

**Introduction**

Rise in urbanization and industrialization in the recent past has led to rapid life style changes. This is having a significant impact on the nutritional and health status of populations, particularly in developing countries. [^1^](#_ENREF_1) Technological development has increased commuting, fast foods, international food chains, home deliveries of most of the products of daily use, television and internet have led to sedentary lifestyle. This is leading to obesity in the population. Most of the chronic diseases like diabetes, cancer, hypertension and cardio-vascular diseases are outcome of the obesity, a consequence of the sedentary lifestyle and unbalanced diets. Food basket have changed from traditional home-cooked meals to junk food further adding to the risk factors and micronutrient deficiencies.[^2^](#_ENREF_2)

While there are biological factors for increased blood glucose, blood pressure, obesity and raised lipids, social determinants and behavioral risk factors such as, tobacco and alcohol use, unhealthy diet, and sedentary lifestyle are leading to malnutrition and chronic diseases.[^3^](#_ENREF_3) These determinants and risk factors operate into a series of critical pathways which result in chronic diseases such as, diabetes, ischemic heart disease, cancers, stroke and chronic lung diseases.[^4^](#_ENREF_4)

Although most chronic diseases are on rise globally, the problem for Asia Pacific region needs to be addressed keeping in mind the socio-cultural epidemiological transitions. The region is struggling to manage communicable diseases and population development issues, simultaneously; it is undergoing drastic changes in the lifestyle as a result of economic development. Increase in chronic diseases, including obesity and diabetes, has grown parallel the growth of urbanization and globalization in the region.[^5^](#_ENREF_5)

About 53 percent of the deaths that occur in India are caused by chronic diseases. The burden of chronic diseases and resultant mortality is expected to increase unless massive efforts are made to prevent and control risk factors of these diseases. Though Government of India has initiated national health programs for the prevention and control of chronic disease, it is at a nascent state of implementation. It is evident that nutritional deficiencies and chronic diseases can only be prevented by addressing the associated risks. Therefore, increasing knowledge of various risk factors among people, such as diet and physical activity becomes an important intervention for prevention and control of the chronic diseases.[^6^](#_ENREF_6)

Once the disease like hypertension and diabetes have occurred then there is no cure and lifelong management is needed. Often these diseases occur at an age where behaviors are already ingrained and taste for unhealthy foods is established. Therefore, to reduce them it is important to “catch them young’’. The young people need to be monitored for their behavior that might be putting them at risk at a later stage in life. School settings, both in rural and urban areas, can be used as a platform for action, where not only students but families of students and teachers can be involved.[^7^](#_ENREF_7)

Behavior change for prevention of chronic diseases mostly comprises of lifestyle modifications. Lifestyle changes refers to adopting healthier eating habits, as increasing fruits and vegetables in the diet and cutting down on the processed foods that are high in sodium and sugar levels along with being physically active. However, to change behaviors, it becomes important to work on the roots causes of behaviors also. Lifestyles and habits are shaped by social conditions, cultural norms and market driven promotional strategies. [^8^](#_ENREF_8)

Countries having policy for healthy foods, low alcohol and tobacco use have been found to be effective in increasing the number of quality years of their population. In the absence of such policies there is needed to change the behaviors along with the lifestyle that people follow. The behaviors are often determined in the childhood based on the lifestyle being followed in the families. The evidence from school-based interventions suggests that schools should address not only adolescents’ behaviors, but the components of the school and community environment that have influence on adolescents ’behaviors and health. Therefore, health promotion interventions need to be developed for school settings to reduce the risk factors of chronic diseases.[^9^](#_ENREF_9)

Most of the chronic diseases have the common risk factors. The intervention on one or more such diseases may also help in preventing other chronic diseases. Therefore, rather than focusing on disease, we are focusing on the prevalence of risk factors and intervention that can alter the lifestyle and behavioral factors. However, it is important to consider the most prevalent chronic diseases; diabetes and hypertension to measure the effect of health promotion intervention.

**REVIEW OF LITERATURE**

The state of knowledge about the prevalence, underlying factors that make people more vulnerable to risky behaviors and interventions to reduce the risky behaviors and chronic diseases was reviewed using MESH words, such as, diabetes and prevalence, hypertension and prevalence, intervention, control, health promotion, chronic diseases burden, health promotion intervention and chronic diseases and school intervention and chronic diseases. Google Scholar and PubMed were comprehensively and extensively searched. Filter was added for review papers only. After reading abstracts, review papers relevant to the topic were selected. Then, research papers on interventions and health promotion strategy were selected. Interventions on all age groups were included in the search.

**Prevalence and Risk Factors of Chronic Diseases**

Chronic diseases are on rise these days and are the largest cause of death in the world. Despite growing evidence of epidemiological and economic impact, the global response to prevent these diseases remain inadequate.[^10^](#_ENREF_10)

Historically, diabetes and hypertension were considered a disease confined to developed countries and affluent people. However, the prevalence of these diseases is rising globally, particularly in developing countries.[^11^](#_ENREF_11) India is currently experiencing an epidemic of type 2 diabetes mellitus (T2DM) and has the largest number of diabetic patients. It is often referred to as the diabetes capital of the world.[^12^](#_ENREF_12) The absolute number of the patients is more in the developing countries due to the large population size.[^13^](#_ENREF_13) Similarly global burden of hypertension will increase to more than 1.56 billion by 2025.[^14^](#_ENREF_14) It is also estimated that the negative health impact and the economic consequences of the hypertension in future will continue to increase in low and middle income countries.[^15^](#_ENREF_15) South Asians are known to have an increased predisposition for Type 2 diabetes.[^16^](#_ENREF_16) Progression of diabetes is known to be more rapid among South Asians and the decline in glycaemic control over time was much more rapid among South Asians when compared to Europeans.[^17^](#_ENREF_17) Hence, it is apparent that diabetes among South Asians represents a significant health concern with differential risk factors and a more aggressive progression than in other ethnic groups.[^16^](#_ENREF_16)

Disease risk factors have their biological as well as social and behavioral origins. Whereas biological factors such as, genes predispose people, the social determinants like education, socio-economic factors, nutrition, housing, sanitation, cultural and ritual practices etc. make people more prone to these diseases. There are some behavior factors that make people more prone to disease and death such as alcohol, tobacco and drug use.[^18^](#_ENREF_18)

Potential reasons that lead to risk factors of various chronic diseases include various reasons, as, stress leading to over-eating, stress could be of various kinds, as stress to fit in peer group and to be accepted socially, of looking good, doing well academically, of class work, financial stress and stress of maintaining personal relationships. Other reasons can be like, no availability of playgrounds or parks in school and around the local area , no street lights in parks, making difficult to walk at dusk and dawn ,no physical education (PE) period in schools or teachers taking up the PE period to teach theoretical subjects, more fast food and junk food options available around the school than healthy food, skipping breakfast or not taking lunch along to schools and work places, watching too much TV or surfing net in leisure time and using bicycles or other vehicles to cover walking distances. [^19^](#_ENREF_19)

Homo sapiens survival during the evolutionary history was dependent on the procurement of food,[^20^](#_ENREF_20)^,^ [^21^](#_ENREF_21) which was dependent on the physical activity. However, food supply was not always consistent. Thus it is contended that our hunter-gatherer ancestors had cycles of feast and famine, punctuated with obligate periods of physical activity and rest. To ensure survival during periods of famine, certain genes evolved to regulate efficient intake and utilization of fuel stores. Such genes were termed "thrifty genes". Some of these genes had functions that were predominantly of glycogen conservation and replenishment. [^22^](#_ENREF_22)

The availability of food and no physical activity eliminated the evolutionarily programmed biochemical cycles emanating from feast-famine and physical activity-rest cycles, which in turn abrogates the cycling of certain metabolic processes, ultimately resulting in various metabolic derangements such as obesity and Type 2 diabetes.[^22^](#_ENREF_22)

Many studies and investigations have been carried out on the metabolism of polyunsaturated fatty acids (PUFAs) in general and specifically on n-3 fatty acids in particular. N-3 fatty acid are essential for normal growth and development and play an important role in the prevention and treatment of various chronic diseases, such as, coronary artery disease, hypertension, diabetes, arthritis, other inflammatory and autoimmune disorders, and cancers.[^23-29^](#_ENREF_23). Human beings have evolved consuming the diet that consisted of equal amounts of n-3 and n-6 essential fatty acids. Over the past hundred years, there has been an enormous increase in the consumption of n-6 fatty acids because of the increased intake of vegetable oils from corn, sunﬂower seeds, safflower seeds, cottonseed, and soybeans.[^30^](#_ENREF_30)

In Western diets, the ratio of n-6 to n-3 fatty acids ranges from <20–30:1 instead of the traditional range of 1–2:1. Some studies indicate that a high intake of n-6 fatty acids shifts the physiologic state to one that is pro-thrombotic and pro-aggregatory, characterized by increases in blood viscosity, vasoconstriction and vasospasm and decreases the bleeding time. N-3 Fatty acids, however, have anti-inﬂammatory, antithrombotic, anti-arrhythmic, hypo-lipidemic, and vasodilatory properties. These beneﬁcial effects of n-3 fatty acids have been shown in the secondary prevention of coronary heart disease, hypertension, type 2 diabetes, and, in few patients with renal disease, rheumatoid arthritis, ulcerative colitis, chronic obstructive pulmonary disease and Crohn disease. Alpha-linolenic acid, found in green leafy vegetables, flaxseed, walnuts and rapeseeds, desaturates and elongates in the human body to EPA and DHA and by itself probably have beneficial effects in health and in the control of chronic diseases. [^30^](#_ENREF_30)

Therefore, there is need to have diet with the food elements that can prevent disease and promote health. Data on human epidemiology has related diet during prenatal and early postnatal nutrition to susceptibility of adults to chronic diseases such as cardiovascular disease, type 2 diabetes, obesity, and some types of cancer. [^31-35^](#_ENREF_31)Nutritional perturbation of epigenetic gene regulation is a likely link between early nutrition and later metabolism and chronic disease susceptibility.[^36^](#_ENREF_36)

Some studies show that subtle nutritional inﬂuences during the development period can inﬂuence adult metabolism. Understanding these particular biological mechanisms underlying such phenomena should enable early life nutritional interventions, or corrective therapies, to prevent chronic diseases in human population. The term “metabolic imprinting” was coined to encompass a subset of adaptive responses to early nutrition, characterized by susceptibility limited to a critical onto-genic period and a persistent effect lasting into adulthood. [^35^](#_ENREF_35)

Therefore, diet is very important, especially in the most rapid growth phase of the life, i.e. adolescence. Not only this, many young people at this stage experiment with various activities that are either health promoting like involvement in sports and other physical activities or health risking, like smoking, taking drugs and consumption of alcohol. These activities lead to nutritional deficiency at the time when it is most important. These behavior are to a very large extent are affected by peer, family and environment.[^37^](#_ENREF_37) To address such behaviors, the involvement of all stakeholders such as parents, teachers is required.[^38^](#_ENREF_38)

The diet, physical activity and substance addiction are dependent on many other factors. Advocating for balanced or healthy diet for people who either do not know or cannot afford will be of no use. Not only this, the risk factors of diseases, such as, diabetes and hypertension are linked to the socio-economic status of the individuals, with those belonging to lower socio-economic classes are more prone to these diseases. In low socio-economic classes also, the impact of these risk factors is more among women compared to their male counterparts.[^39^](#_ENREF_39)

The etiology of diabetes & hypertension is multifactorial like most of the chronic diseases, and there are various causes including nutritional deficiencies, chronic infections, obesity, and chronic non-communicable diseases.[^40-42^](#_ENREF_40)

According to Hawkes et al. (2006) in countries with high income, the prevalence of poor quality diets, obesity and diet-related chronic diseases tends to be higher among groups of lower social-economic status. This trend is now also beginning to emerge in middle-income countries. Obesity starts out as a problem among groups of higher socio-economic status, but as national economies grow, the risk moves towards groups of lower social economic status. [^43^](#_ENREF_43)^,^ [^44^](#_ENREF_44)

Eating behavior is highly complicated and is result of the interplay of multiple inﬂuence across different contexts. At Individual level factors related to food choices and eating behaviors includes cognition, behavior, and biological and various demographic factors. These individual factors can impact food choices through characteristics such as motivation, self-efficacy, expected outcomes, and behavioral capabilities. An environmental context is also related to eating behaviors which includes social environment, physical environment, and macro level environments.[^45^](#_ENREF_45)

The social environment consists of interactions with family, friends, peers, and other members in the community and may impact food choices through mechanisms such as role modeling, social support, and social norms. The physical environment contains the multiple settings where people eat or procure food such as the home, work places, schools, restaurants, and supermarkets. The physical settings within the community that inﬂuence which type foods are available to eat and promote barriers and opportunities that facilitates or hinders healthy eating. Macro level environmental factors have more distal and indirect role but have a substantial and powerful effect on the diet of people. Macro-level factors operating within the larger society includes marketing of food, social norms, food production and distribution systems, agricultural policies, and economic price structures. These four broad levels, inﬂuences, such as. Individual, social environment, physical environment, and macro level environment, all interact, both directly and indirectly, to impact eating behaviors.[^45^](#_ENREF_45)

It is not only availability of food, food habits or eating patterns that lead to chronic disease but physical activity at the optimal level also needs to be ensured. As has already been mentioned that socio-economic and technological development has led to sedentary life of people, it is all the more important that people consciously spend some time on physical activity. Review studies indicate that physical activity has probable beneficial effects on psychological well-being, self-esteem, overweight and obesity, and chronic disease risk factors among young people.[^46^](#_ENREF_46) However, recent cultural changes have engineered physical activity out of the daily lives of humans. Such as, many individuals no longer use manual labor to procure food or shelter. As a result of habitual physical inactivity into incorporation of it as the pattern of daily living, the risks of at least 35 chronic health conditions have increased [^47^](#_ENREF_47)^,^ [^48^](#_ENREF_48). In addition to the diet, the physical activity is equally important in reducing the risk rates of various chronic diseases. Kesaniemi*et al.* (2001) concluded that physical activity is associated with a reduction in all-cause mortality, fatal and non-fatal total cardiovascular disease, and a reduction in the cases of obesity, diabetes mellitus, colon cancer and osteoporosis.[^49^](#_ENREF_49)

Demographic and environmental factors play a very important role in the physical activity among the adolescents. A study suggests that there is significant interaction between season and geographical locations, both these factors play a role in adolescents’ activity levels. Adolescents were more active during summers than during winters, adolescents in urban schools were more active in the winter and adolescents in rural schools were more active in the summer. [^50^](#_ENREF_50)

Tobacco use is a major contributor to deaths from chronic diseases worldwide, and the ﬁndings from the Global Youth Tobacco Survey indicate that current dire warnings of a doubling of the death toll to 10 million deaths per year by 2020 could be a conservative estimate, and the actual toll from tobacco use could be even greater than this estimation.[^51^](#_ENREF_51)

Many interventions have been tried to reduce the risk factors that lead to chronic diseases and deficiency diseases. Most of these are directed to change individual behaviors in different settings. Moreover, most of the interventions are either on changing diet or dietary patterns of individuals or are on physical activity exclusively. There are very few studies on tobacco, alcohol or drugs as risk factors for chronic diseases and intervention to combat these risk factors.[^52^](#_ENREF_52)

**Interventions for Prevention and Control**

Evidence over the past 20 years from a variety of sources, including epidemiological, prospective cohort, and intervention studies, has documented that physical activity, diet, and combined physical activity and diet interventions can mitigate progression of chronic disease and in fact reverse existing disease. [^53^](#_ENREF_53)

Control of chronic disease prevention is most effective if environmental and policy approaches are the earliest focus of change. These approaches are mostly low cost, high reach, and tend to provide supportive environment for later targeted interventions. For example, if one is implementing a community-based program to promote energy balance (healthy eating and physical activity), there is little chance of effectiveness if there is a lack of access to healthy foods or places for activity. This fits well with the World Health Organization’s statement in its Ottawa charter that “Healthy choices need to be the easy choices.” [^54^](#_ENREF_54)^,^ [^55^](#_ENREF_55)

Tailored interventions are designed for individuals based on their unique characteristics, related to the outcome of interest, and derived from an individual assessment. Recognizing the differences between individuals and delivering interventions tailored to the individual could increase the likelihood of sustained self-management of long-term conditions. Ten studies were included in the review. Tailored interventions had no impact on self-management activities such as medication adherence, self-monitoring, exercise, smoking, or diet control. However, tailored interventions were modestly successful in improving specific self-management behaviors of dietary fat intake, levels of physical activity or screening. Studies included in the review generally suffered from compromised methodological issues of inadequately powered sample size, non-blinding of data collection or intervention delivery and inadequate reporting of the randomization process. When cost and resource utilization is taken into consideration, tailored interventions may not be more effective than standard interventions in improving self-management behaviors in individuals with long-term conditions. Future research should explore the effect of robust and resource-optimized tailored interventions on self-management outcomes for long-term conditions with high-quality trials. [^56^](#_ENREF_56)

It has been recognized low adherence to dietary advice can be a major risk to the health and well-being of patients, and greater and good adherence to dietary advice is a critical component in prevention and management of various chronic diseases[^45^](#_ENREF_45). Diet adherence outcomes between an intervention group and a control/usual care group have been reviewed using 38 intervention studies involving 9445 participants. It was found that, 32 out of 123 diet adherence outcomes favored the intervention group, 4 favored the control group and 62 had no signiﬁcant difference between the groups.[^45^](#_ENREF_45)

In a meta-analysis of various interventions used in disease management programs for patients with chronic illnesses, it was found that all studied on interventions were associated with improvements in provider adherence to practice guidelines and disease control. The type and number of interventions varied greatly. Patient qualification was the most commonly used intervention, followed by education of healthcare providers and provider feedback. Most programs used more than one intervention. Provider education, feedback, and reminders were associated with significant improvements in provider adherence to guidelines and with significant improvements in patient disease control. Patient education, reminders, and financial incentives were all associated with improvements in patient disease control. [^57^](#_ENREF_57)

A meta-analysis showed that behavioral treatment interventions have a significant positive effect on adherence in adults with obesity. It provided evidence that behavioral treatment strategies improve adherence to lifestyle intervention programmes in adults with obesity. The findings of this review provide novel insight to clinicians working in obesity and have important implications for lifestyle intervention programme design.[^58^](#_ENREF_58)

These strategies should be routinely incorporated into lifestyle intervention, obesity management and weight loss programmes with the aim of improving engagement and adherence. Further research is required in this area to increase our understanding of the factors that contribute to adherence and how we can better assist adults with obesity. If adherence were improved, treatment effectiveness, health outcomes and the ultimate burden of chronic disease could also be improved. [^58^](#_ENREF_58)

A comprehensive school health project, in [United States](https://en.wikipedia.org/wiki/United_States) of America, the Kansas LEAN School Intervention Project, in Salina and Dighton had four components, three of which were school based: (a) modified school lunches, (b) enhanced nutrition education, and (c) increased opportunities for physical activity. Students' knowledge, skills, and behaviors related to nutrition as well as their physical fitness improved in both Kansas communities. Ultimately, leading to the reduction in risks of chronic diseases among adolescents .[^59^](#_ENREF_59)

In one study, by Prabhushankal *et al.* (2015), eight schools in Chandigarh, India, were given lifestyle intervention for the period of 12 weeks and were assessed for health behaviors, anthropometry and biochemical profile. But, there was no significant change in the biochemical and anthropometric parameters in pre and post intervention. This study also highlighted on the fact that longer duration interventions are required for any significant change in anthropometry and biochemical profile. [^60^](#_ENREF_60)

Yach*et al.*(2005) in the “Go-for-Health Project”, reported that interventions based on organizational change and social learning theory facilitate changes in diet and exercise behavior among elementary school adolescents . Baseline data documented the need for behavior change. Based on chemical analyses, average per meal amounts of total fat and sodium were higher than national recommendations: total fat was 29.3% higher and sodium was 107.4% greater than recommended levels. Observations of students in physical education class revealed adolescents moved through space 50.1% of the time and moved continuously an average of 2.2 minutes per class period. These findings suggest the need for policy and practice changes in the school environment to enable adolescents to engage in more healthful diet and exercise behavior. The key features of ‘Go-for Health’ were organization-level modification of school lunch and physical education was to create an environment supporting healthful diet and physical activity practices, and implementation of classroom instruction based on a well-defined and developed theory consistent with the school environment. The model provided the means of evaluating the effectiveness of combining organizational change and social learning theory strategies. Go-for-Health provides a model for school-based programs for other health behaviors.[^9^](#_ENREF_9)

Some of the studies focusing on the impact of mHealth (Mobile Health) interventions on chronic disease outcomes in LMICs. mHealth was found to have positive impact on processes of care and clinical outcomes. Yet, the effect was modest. In addition, the evidence is still scarce with respect to the effectiveness of different types of mobile interventions to reduce chronic diseases. Further research is needed to assess the effectiveness and cost effectiveness of mHealth strategies, particularly to address hypertension, cancer, and chronic respiratory diseases. [^61^](#_ENREF_61)

A school and community based intervention to influence health behavior and cardiovascular disease (CVD) risk factors in 13 to 15 year-old adolescents were carried out in North Karelia, Finland. The intervention was carried out on two levels: (a) an intensive intervention in two schools and (b) a county-wide intervention in the rest of North Karelia. 851 adolescents, their parents, and their teachers were studied at the outset in 1978 and after the intervention in 1980. The intervention had no effect on blood pressure although there was some reported reduction in the salt consumption in the intervention schools. A relative decrease according to the intervention level was observed among North Karelian adolescents in occurrence of psychosomatic symptoms. The effects were not related to increases in health knowledge or changes in attitudes and did not lead, on an average, to any harmful emotional consequences.[^62^](#_ENREF_62)

In another study from Chandigarh, four schools were selected randomly and 201 students for randomized controlled trial were selected and given lifestyle intervention for 20 weeks for diet and physical activity and primary outcome measures included anthropometric measurements

And the secondary outcomes were biochemical parameters, physical activity and dietary intake. Post intervention there was no significant difference in the anthropometric and biochemical measures. [^63^](#_ENREF_63)

In another study from Kerala, India, a cluster randomized controlled trial was designed for dietary behavior change, the study suggested that male participation is very important for the behavior change intervention and the role of local authorities and community volunteers in making the interventions sustainable in long run. [^64^](#_ENREF_64)

Several components of lifestyle interventions have been associated with improved insulin mediated glucose transport and therein reduce insulin resistance and progression to glucose intolerance. Our findings were generally supportive of the diabetes prevention trials, which suggest that multi-component interventions, including elements of calorie restriction, PA, and behavioral support are most effective in improving glucose tolerance.[^65^](#_ENREF_65)

It is important to ensure that interventions to prevent morbidity and mortality from chronic diseases need to be cost eﬀective and ﬁnancially feasible in countries of low or middle income before recommendations for their scale-up[^66^](#_ENREF_66). Although, the more attention is always given to the influence of the community environment on the various physical activity and dietary behavior[^67-70^](#_ENREF_67), the schools are the most suitable place to base physical activity and healthy nutritional activities to catch young and to have lifetime effect. However, little attention has been given to the role of school environment in influencing this behaviors.[^71^](#_ENREF_71)

The classroom-based, public health approach improved adolescent’s cardiovascular disease risk profiles. It is practical and fairly easy to incorporate into the school day. All adolescents directly receive the potential benefits of the intervention without a risk of labeling. This program can improve health knowledge, habits, and health outcomes of young adolescents at a time when health habits are being formed. In one study, randomized controlled field trial in 12 schools across North Carolina, stratified by geographic reg ion and urban/rural setting, participants were 1274 third and fourth graders. The intervention, taught by regular classroom and physical education teachers, provided all adolescents an 8-week exercise program and 8 weeks of classes on nutrition and smoking. The result of the intervention was that adolescents in the intervention group had significantly greater knowledge and a significant increase in self-reported physical activity than adolescents in the control group. [^72^](#_ENREF_72) There are limited data for structural interventions directed at the social determinants of chronic diseases, including health systems. This is an area that deserves immediate focused attention.[^73^](#_ENREF_73)

The large and increasing burden of chronic diseases can be reduced through systematic and sustained implementation of environmental and policy interventions. Before implementing an array of individual-level programs to prevent chronic diseases, practitioners should consider the power of environmental and policy approaches to set the stage for other interventions.[^74^](#_ENREF_74) However, it is not possible to bring big policy changes but school level policies are possible and implementable and therefore, should be considered.

School setting is chosen as over half of chronic diseases related deaths are associated with behaviors and activities that begin or are reinforced during the adolescence.[^75^](#_ENREF_75) The education sector has the power to reach the local communities also in addition to the students attending the schools or colleges. Benefits from school can reach families and communities. Adolescents can successfully encourage and influence their parents and communities to adopt and maintain healthier lifestyles.[^18^](#_ENREF_18)^,^ [^76^](#_ENREF_76)^,^ [^77^](#_ENREF_77) Interventions carried out in schools have great power to diffuse out and reach great distances and can prove to be beneficial to masses.

**Table:** Review of Literature (Intervention Studies)

| **S.No** | **Author** | **Year** | **Title** | **Journal** | **Objective** | **Intervention** | **Assessment parameters** | **Effect** |
| --- | --- | --- | --- | --- | --- | --- | --- | --- |
| 1. . | Guy S Parcel | 1987 | School Promotion of Healthful Diet and Exercise Behavior: An Integration of Organizational Change and Social Learning Theory Interventions | Journal of School Health | To promote healthy diet and exercise behavior in schools to reduce risk of various diseases | - Modified lunch - Modified physical education periods - Go for health - Classroom instructions | Anthropometric measurements; Mile run test for cardiovascular fitness, Chemical analysis of frequently served meals; frequency of food consumption; aerobic physical activity at and away from school. | Positive for all parameters |
| . | P. Puska | 1982 | The North Karelia youth project: Evaluation of two years of intervention on health behavior and CVD risk factors among 13- to 15-year old children | Preventive medicine | To assess whether health behavior and risk factors  could be influenced by the described intervention program over 2 years. | - Antismoking program - informative materials, skills training, and role plays - Modified school lunch (margarine and skim milk) - Classroom instructions | Biochemical analysis (serum thiocyanate and serum cholesterol), Physiological measurements ( blood pressure), anthropometric measurements | The intervention had no effect on blood pressure although there was some reported reduction in the salt consumption in the intervention schools. A relative decrease according to the intervention level was observed among North Karelian children in occurrence of psychosomatic symptoms. The effects were not related to increases in health knowledge. |
|  | J. Harell | 1996 | Effects of a school-based intervention to reduce cardiovascular disease risk factors in elementary-school children: The Cardiovascular Health in Children (CHIC) study | Journal of Pediatrics | To test a classroom-based intervention to reduce cardiovascular disease risk factors in elementary school children | - Health Education - Physical activity periods | Physiological measurements (cholesterol & blood pressure); anthropometric measurements; aerobic power | Positive |
|  | A. Macaulay | 1997 | The Kahnawake Schools Diabetes Prevention Project: Intervention,  Evaluation, and Baseline Results of a Diabetes Primary Prevention  Program with a Native Community in Canada | Preventive Medicine | To improve healthy eating and encourage more physical activity among elementary school children. | - Healthy food preparation contests - Promotion of traditional food & traditional growing food - Youth Center activities - Walk and Run Club - Health education program - Monthly calendar activity log | One mile run/walk test; Anthropometric measurements; children’s food consumption pattern in previous 7 days; physical activity; self efficacy | Positive for all parameters |
|  | T Prabhushankar | 2015 | Effect of 12-Week Lifestyle Intervention on Behavioral, Anthropometry and Biochemical Profile of School Children in Chandigarh. India. | J Community Med Health Educ | To assess the effect of a 12 weeks lifestyle intervention program on health behavior, anthropometric measures and biochemical profile in selected schools. | - Sensitization session for parents - Physical activity and daily diet recording   (School administration requested for PE period daily) | Biochemical analysis (total cholesterol levels) and anthropometric measurements, behavior modification indicators | Favorable impact on anthropometric and behavioural parameters   - no impact on the biochemical parameters. |
|  | A. Dyson Pamela | 2015 | Successful Up-Scaled Population  Interventions to Reduce Risk Factors for  Non-Communicable Disease in Adults: Results  from the International Community  Interventions for Health (CIH) Project in  China, India and Mexico | PLOS ONE | To develop Interventions to Reduce Risk Factors for  Non-Communicable Disease in Adults frm China, India & Mexico | - “No smoking day” - Healthy snacks at workplaces - “Grow your own” - Physical activity | Anthropometric measurements | Positive for all parameters |
| 7. | J. S. Thakur | 2016 | Impact of 20 Week Lifestyle Intervention Package on Anthropometric Biochemical and BehavioralCharacteristics of Schoolchildren in North India | Journal of Tropical Pediatrics | To examine the impact of a multicomponent lifestyle intervention on weight and body mass index (BMI) of children in a school-based setting. | - Education sessions in schools - Lifestyle diaries | Biochemical analysis (total cholesterol levels) and anthropometric measurements; behavior change indicators | Favorable impact on anthropometric and behavioural parameters   - no impact on the biochemical parameters. |

**Gaps in knowledge**

There are wide differences in the prevalence of chronic diseases in developing and developed countries. These differences could be associated with the socio economic and cultural conditions in which people live, study and work. The interventions which are workable in developed countries may not be workable and effective in developing countries and there might be a need of different interventions in urban and rural areas. The most efficient interventions have occurred at community level for prevention and control of various chronic diseases, whenever the intervention was permanent or maintained for long periods, and relied on the continuous education of community health workers that had a constant interference inside the population covered. It is important that the intervention should be sustainable, otherwise health workers at some point may lose interest, and therefore, it may be worth experimenting to involve a family member who is young to bring change.[^78^](#_ENREF_78)

Literature on chronic diseases like hypertension and diabetes were mostly done on patients and not much is known about interventions among adolescents except for obesity. Less focus is on prevention of chronic diseases and more studies are on the treatment of the diseases. At present there are some indications that adolescents in the upper end of the cholesterol and blood pressure distribution seem to stay there later.[^79^](#_ENREF_79)^,^ [^80^](#_ENREF_80) Thus, not only should prevention be aimed at the whole population, it also may be useful to give special attention to adolescents to reduce the risk factor distribution.[^81^](#_ENREF_81)

Some studies have focused their actions in adolescents, especially on students, because they were more influenced by educational activities of prevention, and the knowledge ac­quired by them would spread more easily to their family and to society.[^49^](#_ENREF_49) But, none of the studies among the students and adolescents have looked into the effect of intervention on behaviors of teachers and parents of the adolescents.

From the previous intervention studies, it is evident that only those interventions are successful in which the researcher is involved in the intervention and it is the direct intervention. The indirect interventions are not that successful and also the intensity and duration of the intervention is very important for it to be a success.

Most of the interventions lack sustainability which reduces its ultimate impact.

**AIM**

To assess the effect of school-based health promotion intervention package among adolescents, their parents and teachers on the risk factors (unbalanced diet, physical inactivity, obesity, use of tobacco & alcohol) of chronic diseases.

**Objectives of the study are to:**

1. Measure behavioural (unbalanced diet, physical inactivity, and tobacco & alcohol use), anthropometric (height, weight, waist circumference, hip circumference, mid-upper arm circumference, subscapular and triceps skinfold thickness), physiological (blood pressure) and biochemical (fasting plasma glucose and urinary sodium excretion levels from 24-hour and spot urine samples) risk factors of chronic diseases among adolescents, their parents and teachers
2. Develop and implement a health promotion intervention package of six-month duration in school settings
3. Determine the effect of the intervention package on behavioural, anthropometric (height and weight), physiological (blood pressure), and biochemical (urinary sodium excretion levels from spot urine samples) risk factors of chronic diseases among adolescents

**HYPOTHESIS**

**Null hypothesis:**

School-based health promotion intervention will not lead to any change in the dietary intake of salt (g/day), sugar (g/day), fruits (g/day), vegetables (g/day), physical inactivity (%), tobacco and alcohol use (%) among adolescents.

**Alternate hypothesis:**

School-based health promotion intervention will decrease the dietary intake of salt (g/day) and sugar (g/day), increase the intake of fruits (g/day) and vegetables (g/day), and decrease the physical inactivity (%) and tobacco and alcohol use (%) among adolescents.

**Methodology**

**Study area**

The study area will be the urban area of **Union Territory of Chandigarh, India.**

Chandigarh is the planned city of the India. The population of the city is 960,787 with the sex ratio of 818 females per 1000 males. Literacy rate of the city is 86.77%. It has 188 schools out of which 115 are government schools and 7 are government aided schools.

| Schools | Government | Recognized private schools |
| --- | --- | --- |
| Nursery School | 1 | ____ |
| Primary Schools | 8 | 4 |
| Middle Schools | 13 | 13 |
| High Schools | 53 | 22 |
| Senior Secondary Schools | 40 | 34 |
| Total schools | 115 | 73 |

***Class wise enrollment of Govt. Schools of Upper Primary Level (Class 8^th^) over the years:***

| **Year** | **Number of students enrolled** |
| --- | --- |
| 2013-2014 | 13,303 |
| 2014-2015 | 14,047 |
| 2015-2016 | 13,776 |
| 2016-1017 | 14,036 |

**Study setting**

Study setting will be schools (government) of Chandigarh UT.

**Population**

The study will be conducted among students, their parents and teachers. As the study focuses mainly on the students and reaches out to their parents & teachers, the study population will be the individuals in the age group of 11-16 yrs for students, their parents & teachers up to age of 65 years.

**Study Design**

Study design is Cluster Randomized Controlled Trial. Each school will be one cluster.

Schools will be randomized to intervention and control group and net changes in the knowledge and practices related to chronic disease risk factors among students, their parents and teachers will be computed by measurements before starting the health promotion intervention and after implementation of the intervention in both intervention and control clusters.

Research approach will be mixed methods: It will have both qualitative & quantitative data. Quantitative data will be collected using validated tools. Qualitative data will be collected by carrying 10 focused group discussions with students, their parents and teachers separately to understand their perception about risk factors and lifestyle behaviors (2 with male teachers, 2 with female teachers, 2 with female students, 2 with male students and 2 with parents in government schools).

**Sample size**

A total of 12 clusters were calculated based on the formula as shown below. Six clusters in intervention arm and six in control arm. As most of the schools in general have strength of 30 students in each section, the size of cluster (m) was taken to be 30 each from a randomly selected section of class 8^th^. Sample size for each indicator (risk factors: Unbalanced diet, physical inactivity, tobacco use and alcohol use) was calculated separately by using data from previous studies.

For quantitative data the sample size will be the same, i.e., 6 clusters in intervention arm & 6 in control arm for students, their parents & teachers in total. For qualitative data the sample size will be max up to 8 participants for each of the focus group discussion.

**Formula used:**

**c = 1 + f [ π_0_(1 - π_0_)/m + π_1_(1 - π_1_)/m + k^2^ (π_0_^2^ + π_1_^2^) ] / (π_0_ - π_1_)^2^**

Where,

- c is the number of clusters per treatment arm
- f = 7.84 for 5% type I error and 80% power
- π_1_ is the expected proportion in the intervention arm
- π_0_ is the expected proportion in the control arm
- m is the number of individuals in each cluster (assumed equal in all clusters)
- k is the coefficient of variation in the (true) rates or proportions between clusters in each treatment arm

**Sample size based on the risk factors**

| **Risk Factor** | ***π_0_*** | ***π_1_*** | ***K*** | ***C*** |
| --- | --- | --- | --- | --- |
| Inadequate fruits and vegetable intake^82^ | 0.852 | 0.596 | 0.025 | 12.2 |
| Excess salt intake^83^ | 0.223 | 0.162 | 0.094 | 12.1 |
| Sugar intake (g/day)^84^ | 0.507 | 0.355 | 0.041 | 12.5 |
| Physical inactivity^85^ | 0.232 | 0.162 | 0.099 | 12.1 |
| Tobacco use^85^ | 0.049 | 0.034 | 0.163 | 11.4 |
| Alcohol use^86^ | 0.345 | 0.242 | 0.057 | 12.4 |
| Total clusters selected: Twelve | | | | |

Schools will be selected randomly for control and intervention groups (based on the consent) from the list of randomly selected schools.

**Sampling Framework**

***Schools:***

There are total 188 schools in Chandigarh, of which 115 are government and 73 are private. Twenty-four schools (double than the required number of twelve schools) will be randomly selected from the list of 115 public schools functioning in Chandigarh city. Administrators of these 24 schools will be approached from the list sequentially for obtaining their consent. After receiving consent from the first twelve schools, the list will be closed. From within the 12 selected schools, 12 clusters will be selected randomly using the following procedure.

***Adolescents***

A number will be assigned on folded paper slips for all the sections of the 8^th^ grade. Later, a slip will be randomly selected from the shuffled lot by a person other than the researcher. The section numbered in the slip will be then be selected as the cluster for the study. The above-mentioned method will be applied to select a section of 8^th^ grade in each of the selected twelve schools. Adolescents studying in 8^th^ grade are between 10-16 years of age. All students from class who give the consent to be part of the research study will be involved in the study.

***Parents & teachers***

Parents (or guardians) & teachers (class teacher, subject teachers, physical education teacher, counselor, mid-day meal in-charge (wherever, applicable) and medical in charge) will be involved in the research study, only the parents (or guardians) and teachers who give consent will be part of research study. Either mother or the father of the child will be involved in the research study based on their time availability.

**Intervention Development**

Interventions will focus on following risk factors leading to chronic diseases:

- Poor dietary habits
- High salt intake
- High sugar intake
- Eating junk and fast food
- Overeating
- Physical inactivity
- Sitting for long hours
- Very less walking and no form of exercise
- Tobacco smoking & chewing
- Alcohol

As the major reasons for the most of the chronic diseases are poor diet and physical inactivity. It is important to understand what social, environmental and behavioral factors affect the diet and physical activity and also what other factors such as tobacco & alcohol may lead to or contribute to risk factors of various chronic diseases.

**Methods for Objective 1**

Quantitative data will be used to identify behavioral risk factors.

A baseline survey will be carried out where, demographic details of each participant such as name, age, sex, education, occupation, number of family members, annual income, and knowledge and practices about the risk factors of chronic diseases will be collected from students, parents and teachers. Anthropometric measurements, such as, height and weight, BMI, waist circumference, hip circumference, waist-hip ratio, skin-folds (triceps and sub-scapular), mid- upper arm circumference and maximum hand grip strength will be taken before and after intervention. This will be one of the parameter to assess the effectiveness of the intervention. Hypertension will be checked for students, parents and teachers in pre & post intervention in both intervention and control arm.

**Prevalence will be measured for different set of audience as presented below:**

| **Measurements (Physiological)** | **Audience** |
| --- | --- |
| Blood Pressure | Students, their parents & teachers |
| **Measurements (Anthropometric)** | **Audience** |
| 1. Height 2. Weight 3. BMI 4. Waist circumference 5. Hip circumference 6. Waist-Hip Ratio 7. Mid upper arm circumference 8. Skin-folds  A) Triceps  B) Sub-scapular 9. Maximum hand grip strength | Students, their parents & teachers |
| **Measurements (Biochemical)** | **Audience** |
| 1. Fasting blood samples (plasma glucose levels and lipid levels) 2. 24-hour and spot urine samples (24-hour excretory sodium levels) | Students, their parents & teachers |

**Procedure for measurements**

**Anthropometric measurements**

1. Height
   Make the participant stand straight on the floor board of the stadiometer with his or her back to the vertical backboard of the stadiometer. The weight of the participant should be evenly distributed on both the feet. The heels of the feet should be placed together with both heels touching the base of the vertical board. The feet should be pointed slightly outward at a 60 -degree angle (see Exhibit 3-1). If the participant has knock knees, the feet should be separated so that the inside of the knees are in contact but not overlapping. The buttocks, scapulae, and head are positioned in contact with the vertical backboard. It may not be possible for some children and most adults to place their heels, buttocks, scapulae and the posterior aspect of the head against the backboard while maintaining normal stature. Such participants should be positioned so that only the heels and buttocks are in contact with the vertical board, and the body is positioned vertically above the waist. The arms should hang freely by the sides of the trunk with palms facing the thighs. The participant should be asked to inhale deeply and to stand fully erect without altering the position of the heels. The participant’s head should be maintained in the Frankfort Horizontal Plane position while the examiner lowers the horizontal bar snugly to the crown of the head with sufficient pressure to compress the hair. Hair ornaments, buns, braids, etc. must be removed to obtain an accurate measurement. The bar is locked in place and measurement to be recorded by the recorder to the nearest 0.1 cm.
2. Weight

Participant is asked to remove their heavy outer garments (jacket, coat, throusers, skirts, etc.) and shoes. If participant refuse to remove trousers or skirt, at least make them empty their pockets and record the fact in the data collection form. The participant stands in the centre of the platform, weight distributed evenly to both feet. Standing off-centre may affect measurement. The weight is recorded to the resolution of the scale (the nearest 0.1 kg or 0.2 kg).

1. Waist circumference

When participant is in is in a standing position. The examiner stands behind the participant and palpates the hip area for the right iliac crest (see Exhibit 3-6). The examiner marks a horizontal line at the high point of the iliac crest and then crosses the line to indicate the midaxillary line of the body. The pants and underclothing of the participant must be lowered slightly for the examiner to palpate directly on the hip area for the iliac crest. The examiner then stands on the participant’s right side and places the measuring tape around the trunk in a horizontal plane at this level marked on the right side of the trunk. The recorder walks around the participant to make sure that the tape is parallel to the floor and that the tape is snug, but does not compress the skin. The measurement is made at minimal respiration to the

nearest 0.1 cm.

1. Hip circumference

The participant should be standing erect with feet together and weight evenly distributed on both feet. The examiner squats on the right side of the participant and places the measuring tape around the buttocks. The tape is placed at the maximum extension of the buttocks . The recorder then adjusts the sides of the tape and checks the front and sides so that the plane of the tape is horizontal. The zero end of the tape is held under the measurement value. The tape is held snug but not tight.

1. Triceps skinfold

The participant should be standing erect with feet together, shoulders relaxed and the arms hanging freely at the sides. The examiner stands behind the participants’s right side. The point on the posterior surface of the right upper arm is located in the same area as the marked midpoint for the upper arm circumference. A fold of skin and subcutaneous adipose tissue is grasped gently with thumb and fingers approximately 2.0 cm above the marked level with the skinfold parallel to the long axis of the arm. The jaws of the calipers are placed at the marked level, perpendicular to the length of the fold, and the skinfold thickness is measured to the nearest 0.1 mm while the fingers continue to hold the skinfold.

1. Subscapular skinfold thickness

The participant should be standing erect with shoulders and arms relaxed at the side. The examiner palpates for the inferior angle (or triangle portion) of the right scapula. The examiner makes a (+) on the inferior angle of the scapula with the cosmetic pencil marker. The examiner grasps a fold of skin and subcutaneous adipose tissue directly below (1.0 cm) and medial to the inferior angle. The skinfold forms a line about 45 degrees below the horizontal extending diagonally toward the

right elbow. The jaws of the caliper are placed perpendicular to the length of the fold about 2.0 cm lateral to the fingers with the top jaw of the caliper on the mark over the inferior angle of the scapula. The skinfold thickness is measured to the nearest 0.1 mm while the fingers continue to hold the skinfold.

1. Mid-upper arm circumference

Wrap the measuring tape around the arm at the level of the upper arm mid-point mark. Position the tape perpendicular to the long axis of the upper arm. Pull the two ends of the overlapping tape together so that the zero end sits below the measurement value and the result lies on the lateral aspect of the arm (not the posterior surface). Check that the tape fits snug around the arm but does not compress the skin. Take the measurement to the nearest 0.1 cm.

1. Maximum hand grip strength

Ask the participant to squeeze the dynamometer with as much force as possible, being careful to squeeze only once for each measurement. Three trials should be made with a pause of about 10-20 seconds between each trial to avoid the effects of muscle fatigue. Record the result of each trial to the nearest pound or kilogram. If the difference in scores is within 3 kgs., the test is complete. If the difference between any two measures is more than 6.6 lbs. or 3 kgs., then repeat the test once more after a rest period. Use the best 3 measurements (ie. the highest three) in your data report.

**Physiological measurements**

Blood pressure

Insert the air plug into the air jack. The instrument should be on a level with the heart of the participant. Put the participant’s arm through the cuff loops. Position the arm correctly – The bottom edge of the cuff should be 1 or 2 cm above the elbow. Marker (arrow under tube) is centered on the middle of inner arm. Close the Velcro fastener when the cuff snugly encircles upper arm. Press the START / STOP button. Note down the reading of systolic, diastolic pressure, pulse rate Take the reading again five minutes later and note the readings and remove cuff. Take the average of three BP readings to get the BP of the individual

**Methods for Objective 2:**

**Intervention package development**

The intervention package will be developed to change behavior of students, their parents and teachers. Therefore, the age group for measuring change in knowledge and practices will be 11-16 years for students & up to 65 years for parents & teachers.

Qualitative data will be collected by carrying 10 focused group discussions with students, their parents and teachers separately to understand their perception about risk factors and behaviors as well as about hypertension and diabetes.

**Phase 1 (Social assessment) –** Knowledge and practice assessment

**Phase 2-4 (Health sector assessment) -** Epidemiological, behavioral, environmental and motivational assessments.

**Phase 5-7 (Political assessment) –** Intervention, implementation & evaluation.

To develop a successful health promotion intervention for a community/population/group, it is important to know the demands and requirements of the people of the group. For health promotion planning there is need to widen the overlapped area between public perceived needs, actual needs and resources.

**Figure 1**: Health promotion planning model.

To achieve this, precede-proceed model will be followed and planning tool for health promotion intervention will be based on it.

**Phase 5 Phase 4 Phase 3 Phase 2 Phase 1**

Administrative and policy assessment Educational and ecological assessment Behavioral and environment assessment Epidemiological assessment Social assessment

Behavior and lifestyle

Predisposing factors

HEALTH PROMOTION

Quality of life

Health

Health education

Reinforcing factors

Environment

Enabling factors

Policy regulation organization

**Phase 6 Phase 7 Phase 8 Phase 9**

Implementation Process evaluation Impact evaluation Outcome evaluation

**Figure 2**: Precede-proceed model of Health Promotion.^87^

The social change approach will be applied. The first step will be to diagnose the various problems that affect the quality of life of the group and its members, their strengths and weaknesses, their resources and their willingness to change. This will be achieved by conducting various Focused Group Discussions (FGD’s) with the students.

Secondly, the epidemiological, behavioral, and environmental diagnosis will be done to know the physical and social actions that lead to the certain behavior and lifestyle and also the behavior of individuals (students) and actions of decision-makers that affect the behavior of the individuals at risk. This will help in framing more clear objectives and planning for intervention. This will be achieved with the help of questionnaires.

Then, the administrative and organizational concerns will be addressed prior to program implementation. After that intervention implementation, the process of the intervention implementation, its outcomes will be evaluated respectively.

**Intervention Implementation**

Awareness about the risky behaviors and will be created by group discussions during parents & teacher meeting to be held once in every three months. Following WHO Guidelines for Physical Activity and Diet will be followed while developing health promotion interventions among students.

WHO guidelines of physical activity for young people, aged 5-17 will be used for the intervention. Recommended levels of physical activity for adolescents are as follows.

For adolescents and young people, physical activity includes play, games, sports, transportation, chores, recreation, physical education, or planned exercise, in the context of family, school, and community activities.

In order to improve cardio-respiratory and muscular fitness, bone health, and cardiovascular and metabolic health biomarkers:

- Adolescents and youth should accumulate at least 60 minutes of moderate- to vigorous-intensity physical activity daily.
- Amounts of physical activity greater than 60 minutes provide additional health benefits.
- Most of the daily physical activity should be aerobic. Vigorous-intensity activities should be incorporated, including those that strengthen muscle and bone*, at least 3 times per week.

*For this age group, bone-loading activities can be performed as part of playing games, running, turning or jumping.

**Diet**

The exact make-up of a diversified, balanced and healthy diet will vary depending on individual needs (e.g. age, gender, lifestyle, degree of physical activity), cultural context, locally available foods and dietary customs. But basic principles of what constitute a healthy diet remain the same.

Diet evolves over time, being influenced by many factors and complex interactions. Income, food prices (which will affect the availability and affordability of healthy foods), individual preferences and beliefs, cultural traditions, as well as geographical, environmental, social and economic factors all interact in a complex manner to shape individual dietary patterns. Therefore, promoting a healthy food environment, including food systems which promote a diversified, balanced and healthy diet, requires involvement across multiple sectors and stakeholders, including government, and the public and private sector.

Intervention to the students in the schools will be given four times in a week with the help of champions, their buddies and the teachers of the selected schools.

***Intervention package for adolescents will include:***

1. Written information on the importance of different exercises and morning walk.
2. Students will be motivated for recommended level of salt and sugar consumption. Specific activities will be developed to motivate them.
3. Cheaper and healthier eating options available to them will be worked out in classroom settings.
4. Mid-day meal program will be checked for salt and sugar use. Teachers and meal providers will be guided for using recommended levels for salt and sugar.

***Intervention on parents & teachers***

1. Face to face interactions will be held two times during the period of intervention with the parents to brief and debrief them on what is being conveyed to students.
2. Information packages, such as, pamplets, spoons will be sent home through adolescents.
3. Facilitation for behavior change will be provided to parents when needed.
4. Class teachers will be involved in all the activities being carried out with students and parents.

**Detailed intervention plan:**

Physical activity sessions will be included with fun activities to keep students interested in the activity. Two students from each class (one monitor/ champion and one buddy) will be selected and trained for specific exercises. Exercises will be done for 45 minutes for 4 days in each week for 6 months. 45 minutes exercise schedule will consist of half an hour low intensity physical activity, such as, walking and stretching and another 15 minutes of vigorous exercises, such as cardio, consisting of brisk walking, running, and jumping jack and jumping ropes. Trained students, under the supervision of the teachers will lead the exercise classes.

| **Communication objectives** | **Audience** | **Barriers** | **Opportunities** | **Activities** | **Person responsible** | **Frequency & time in one cluster**  **(six month)** | **Channel of communication** |
| --- | --- | --- | --- | --- | --- | --- | --- |
| - To promote the recommended levels of salt, sugar, fruits and vegetable intake, and physical activity and reduction in the use of tobacco and alcohol - To manage hypertension and diabetes by discussing the importance of medicine adherence | Teachers: Teachers in direct interaction with randomly selected clusters (sections of 8^th^ grade) | Different class schedules of the teachers | School hours are the best time to interact with teachers in groups | Educational Sessions with teachers keeping communication objectives in focus | Researcher | Four sessions of thirty minutes during intervention period | Verbal (2-way communication) |
|  | Parents of adolescents from randomly selected clusters | Not all parents are available at the time of the parents-teachers meeting in schools | - Utilising home environment for promoting healthy diets by educating parents (as mostly, mothers are the food makers of the house) on the harmful effects of excessive intake of salt & sugar and less consumption of fruits and vegetables - The Parents Teacher Association (PTA) meetings may be used to interact with parents in school settings | Educational Sessions with parents keeping communication objectives in focus | Researcher | Four sessions of thirty minutes during intervention period | Verbal (2-way communication) |
|  | *Primary audience:* Adolescents of the randomly selected clusters (sections of 8^th^ grade)  *Secondary audience:* Parents & teachers of adolescents | - Vendors outside school premises - Unhealthy food options in the school canteens - Study load (tuitions after school hours & homework) - Stringent school time-table | - To catch adolescents before risky behaviours start developing - Using schools as a platform for promoting healthy behaviours | Educational classes and classroom discussions | Researcher | 30 minutes session every fortnight | Verbal (2-way communication) &  Digital audio-  visual method (PowerPoint presentation using the laptop) |
|  |  |  |  | Physical activity sessions | Researcher/ physical education teachers | Four sessions every week with each session of 30 minutes | Verbal (2-way communication) |
|  |  |  |  | Other interactive activities: Poster making competition & grow your own herbs | Researcher | Two sessions during intervention period with each session of about 45 minutes | Verbal (2-way communication) |
|  |  |  |  | Peer-to-peer education for reduction in the use of tobacco and alcohol | Researcher | One session every month with each session of 15 minutes | Verbal (2-way communication) |

**Behavior Change Communication Matrix- Intervention fr promoting recommended level of salt, sugar, fruits and vegetable intake along with increase in physical activity and reduction in use of tobacco and alcohol in public schools of Chandigarh**

Purpose of the Intervention: To promote positive changes in attitudes and behavior related to salt, sugar, fruits and vegetable intake, increase in physical activity and reduction in use of tobacco and alcohol among adolescents, their parents & teachers.

| **S.No** | **Risk Factors** | **Goal** | **Activities** |
| --- | --- | --- | --- |
|  | **Unbalanced dietary habits** | Recommended level of salt & sugar consumption  Recommended level of fruits and vegetable intake | Goal settings: ***Students***   - To learn how to measure the daily salt & sugar they are consuming in grams. - To remind everyone at home every day to consume less of salt & sugar. - To remove table salt - To stop the use of pickles and sauces - To stop junk food binging - To consume fresh seasonal fruits and vegetables daily   ***Parents***   - To measure the daily salt & sugar they are consuming and using for preparing food with the help of ***measuring tea spoons***.   ***Teachers***   - To measure the daily salt & sugar they are consuming and using for preparing food with the help of ***measuring tea spoons***. - To eat dinner two hours prior sleeping - To walk for 45-60 minutes daily - To take medicine regularly and get regular check-ups if hypertensive and diabetic and regular intake of folic acid tablets if anemic. - To make homemade sauces & pickles instead of consuming ready-made sauces and pickles. - To make fresh vegetables every day and consume fruits everyday |
|  | **Physical inactivity** |  | Goal settings:  ***Students***   - 45 minutes of less to moderate intensity exercise, at least 4 days a week   ***Parents***   - To walk for 45-60 minutes daily - To motivate children for outdoor activities   ***Teachers***   - To walk for 45-60 minutes daily - To motivate children for outdoor activities - To supervise physical activity classes efficiently |
|  | **Tobacco & alcohol use** | To create awareness about the ill effects of tobacco & alcohol | - Knowledge about the ill effects of tobacco & alcohol - To say no to tobacco & alcohol - To ask elders to not smoke or drink in front of you - Harmful effects of passive smoking - To help elders to reduce and stop smoking & drinking |

**Micro-planning of Intervention**

| **S.No** | **Focus of Intervention** | **Goal** | **Goal settings:** | **Activities** | **Time** | **Frequency in one cluster** |
| --- | --- | --- | --- | --- | --- | --- |
|  | **Unbalanced dietary habits** | To reduce salt & sugar consumption  To increase fruits and vegetable intake | ***Students***   - To learn how to measure the daily salt & sugar they are consuming in grams. - To remind everyone at home every day to consume less of salt & sugar. - To remove table salt - To stop the use of pickles and sauces - To stop junk food binging - To consume fresh seasonal fruits and vegetables daily   ***Parents***   - To measure the daily salt & sugar they are consuming and using for preparing food with the help of ***measuring tea spoons***.   ***Teachers***   - To measure the daily salt & sugar they are consuming and using for preparing food with the help of ***measuring tea spoons***. - To walk for 45-60 minutes daily - To take medicine regularly and get regular check-ups if hypertensive and   diabetic and regular intake of folic acid tablets if anemic.   - To make homemade sauces & pickles instead of consuming ready-made sauces and pickles. - To make fresh vegetables every day and consume fruits everyday | ***Students***   - Presentation - Activities - Discussions   ***Parents & teachers***   - Group discussions with parents and teachers | Approx. 30 minutes  30 minutes | One session every week  One session with parents and one with teachers in the duration of six months |

|  | **Physical inactivity** | To increase the level of physical activities and be more active | ***Students***   - 30 minutes of less to moderate intensity exercise, at least 4 days a week   ***Parents***   - To walk for 45-60 minutes daily - To promote outdoor activities among children   ***Teachers***   - To walk for 45-60 minutes daily - To promote outdoor activities among children - To supervise physical activity classes efficiently | ***Students***   - Physical education classes with the help of physical education teacher (any outdoor games like basketball, volleyball, cricket etc. as per the choice of the students)   ***Parents & teachers***   - Group meeting with parents and teachers | 30 minutes  30 minutes | 4 days/ week  One session with parents and one with teachers in the duration of six months |
| --- | --- | --- | --- | --- | --- | --- |
|  | **Tobacco & alcohol use** | To create awareness about the ill effects of tobacco & alcohol | - Knowledge about the ill effects of tobacco & alcohol - To say no to tobacco & alcohol - To ask elders to not smoke or drink in front of you - Harmful effects of passive smoking - To help elders to reduce and stop smoking & drinking | ***Students***   - Peer to peer education   ***Parents & teachers***   - Group meeting with parents and teachers for breaking various myths | 10-15 minutes  30 minutes | one session every month  One session with parents and one with teachers in the duration of six months |
|  | **Management of Hypertension and Diabetes** | To create awareness about methods to manage | - To help adults or anyone near them to manage these diseases - Behavioural modifications- Diet, physical activity, tobacco and alcohol use - Importance of medicine adherence | ***Students, parents and teachers***  ***Classroom discussion*** | 30 minutes | One session, separately with students, parents and teachers in the duration of six months |

**Methods for Objective 3:**

Effectiveness of the intervention will be measured using following indicators in the baseline and end line sample survey among adolescents:

Before the intervention, perceptions, knowledge and practice of case and control group adolescents will be assessed for the chronic diseases and their risk factors with the help of a questionnaire. This will help in measuring the extent of behavior change. This questionnaire will be used as one parameter to assess the effectiveness of the intervention as the same questionnaire will be used after the completion of the intervention. This will help in assessing the improvement of the perception, knowledge and practices.

For objective measurements, anthropometric measurements, such as, height and weight, along with physiological measurement (blood pressure), biochemical measurement (24-hour excretory sodium through spot urine samples) will be taken before and after intervention. This will be the second parameter to assess the effectiveness of the intervention. The net changes in primary and secondary outcome measures among parents and teachers will be considered as exploratory analysis,

| **Intervention** | **Impact** | |
| --- | --- | --- |
|  | **Direct** | **Indirect** |
| Diet | Participant | Family |
| Physical activity | Participant | Family |
| Alcohol | Participant | Family |
| Smoking | Participant | Family |

**Table:** Impact of different interventions on the participants and their families.

| **Expected outcome of Intervention** |
| --- |
| 1. Increase in knowledge about various chronic diseases and their risk factors 2. Positive response for behavior for diet and physical activity 3. Increase in normal BMI and waist-hip ratio 4. Reduction in risk factors of chronic diseases, such as, decrease in salt and sugar intake and increase in iron intake. 5. Reduction in the consumption of junk food. 6. Adherences to the medicine among those having chronic diseases. 7. Change in stage of behavior |

**Table:** Expected outcomes of intervention package

**Statistical Analysis**

Descriptive statistics such as mean, standard deviation, student’s t-test, will be done separately for both the sexes. Chi-square test will be done for categorical variables. Change in various indicators will be computed in intervention and controls. Multivariable regression will be done to measure the effect of intervention after controlling for the confounding variables, if any. All the statistical analysis will be done using Statistical Package for Social Sciences (SPSS) or R.

**Ethical Considerations**

Permission for conducting the study is taken from Institute’s Ethical Committee (INT/IEC/2018/000082; Date: 22/ 01/2019) and the study is registered under the Clinical Trial Registry of India (CTRI/2019/09/021452; 30/09/2019). During this study the participant’s data will be kept confidential. This study will not cause any harm to participants and they can refuse to participate in the study at any time.

**References**

1. Who J, Consultation FE. Diet, nutrition and the prevention of chronic diseases. World Health Organ Tech Rep Ser. 2003;916.

2. Allender S, Foster C, Hutchinson L, Arambepola C. Quantification of urbanization in relation to chronic diseases in developing countries: a systematic review. Journal of Urban Health. 2008 Nov 1;85(6):938-951.

3. World Health Organization. Global status report on non-communicable diseases 2010. Geneva: WHO; 2011.

4. World Health Organization. Preventing chronic diseases: A vital investment.WHO global report. 2005. Geneva: WHO; 2005.

5. Low WY, Lee YK, Samy AL. Non-communicable diseases in the Asia-Pacific region: Prevalence, risk factors and community-based prevention. International Journal of Occupational Medicine and Environmental Health.2015 Jan 1; 28:1-7.

6. Lopez AD, Mathers CD, Ezzati M, Jamison DT, Murray CJL. Global and regional burden of disease and risk factors, 2001: systematic analysis of population health data. Lancet 2006; 367:1747–1757.

7. Brownson RC, Haire-Joshu D, Luke DA. Shaping the context of health: a review of environmental and policy approaches in the prevention of chronic diseases. Annu.Rev. Public Health. 2006 Apr 21;27:341-370.

8. Willett WC, Koplan JP, Nugent R, Dusenbury C, Puska P, Gaziano TA. Prevention of chronic disease by means of diet and lifestyle changes.

9. Parcel GS, Simons‐Morton BG, O'Hara NM, Baranowski T, Kolbe LJ, Bee DE. School promotion of healthful diet and exercise behavior: an integration of organizational change and social learning theory interventions. Journal of School Health. 1987 Apr 1;57(4):150-6.

10. Yach D, Hawkes C, Gould CL, Hofman KJ. The global burden of chronic diseases: overcoming impediments to prevention and control. JAMA. 2004 Jun 2;291(21):2616-2622.

11. Wild S, Roglic G, Green A, Sicree R, King H: Global Prevalence of Diabetes. Diabetes Care 2004; 27:1047–1053.

12. International Diabetes Federation. Diabetes Atlas, 3rd ed., Brussels: International Diabetes Federation; 2006.

13. Kearney PM, Whelton M, Reynolds K, Muntner P, Whelton PK, He J. Global burden of hypertension: analysis of worldwide data. Lancet 2005; 365:217–223.

14. Kearney PM, Whelton M, Reynolds K, Muntner P, Whelton PK, He J. Global burden of hypertension: analysis of worldwide data. Lancet 2005; 365:217–223.

15. Lim SS, Vos T, Flaxman AD, Danaei G, Shibuya K, Adair-Rohani H, et al. A comparative risk assessment of burden of disease and injury attributable to 67 risk factors and risk factor clusters in 21 regions, 1990–2010: a systematic analysis for the Global Burden of Disease Study 2010. Lancet 2012; 380:2224–2260.

16. Jayawardena R, Ranasinghe P, Byrne NM, Soares MJ, Katulanda P, Hills AP. Prevalence and trends of the diabetes epidemic in South Asia: a systematic review and meta-analysis. BMC Public Health. 2012 May 25;12(1):380.

17. Mukhopadhyay B, Forouhi NG, Fisher BM, Kesson CM, Sattar N. A comparison of glycaemic and metabolic control over time among South Asian and European patients with Type 2 diabetes: results from follow‐up in a routine diabetes clinic. Diabetic Medicine. 2006 Jan 1;23(1):94-98.

18. Miller GE, Chen E, Parker KJ. Psychological stress in childhood and susceptibility to the chronic diseases of aging: moving toward a model of behavioral and biological mechanisms. Psychological Bulletin. 2011 Nov;137(6):959.

19. Muennig P. The body politic: the relationship between stigma and obesity-associated disease. BMC Public Health. 2008 Apr 21;8(1):128.

20. Åstrand PO. Textbook of work physiology: physiological bases of exercise. Human Kinetics; 2003.

21. Cordain L, Gotshall RW, Eaton SB. Physical activity, energy expenditure and fitness: an evolutionary perspective. International Journal of Sports Medicine. 1998 Jul;19(05):328-35.

22. Chakravarthy MV, Booth FW. Eating, exercise, and “thrifty” genotypes: connecting the dots toward an evolutionary understanding of modern chronic diseases. Journal of Applied Physiology. 2004 Jan 1;96(1):3-10.

23. Simopoulos AP. Omega-3 fatty acids in health and disease and in growth and development. The American Journal of Clinical Nutrition. 1991 Sep 1;54(3):438-463.

24. Simopoulos AP. Health effects of [omega] 3 polyunsaturated fatty acids in seafoods. In International Conference on the Health Effects of [omega] 3 Polyunsaturated Fatty Acids in Seafoods 1990: Washington, DC,Karger; 1991.

25. Simopoulos AP. Summary of the NATO advanced research workshop on dietary omega 3 and omega 6 fatty acids: biological effects and nutritional essentiality. The Journal of Nutrition. 1989 Apr;119(4):521-528.

26. Simopoulos AP. Health effects of [omega] 3 polyunsaturated fatty acids in seafoods. In International Conference on the Health Effects of [omega] 3 Polyunsaturated Fatty Acids in Seafoods 1990: Washington, DC) 1991.Karger.

27. Simopoulos AP. Essential fatty acids in health and chronic disease. The American Journal of Clinical Nutrition. 1999 Sep 1;70(3):560s-9s.

28. Galli C, Simopoulos AP, Tremoli E. Effects of fatty acids and lipids in health and disease. World Review of Nutrition and Dietetics (. 1994;76.).

29. Simopoulos AP, Salem N. Fatty acids and lipids from cell biology to human disease. Lipids-Chicago-American Oil Chemists Society. 1996 Mar 1;31:S-1.

30. Frankel S, Gunnell DJ, Peters TJ, Maynard M, Smith GD. Childhood energy intake and adult mortality from cancer: the Boyd Orr Cohort Study. British Medical Journal. 1998 Feb 14;316(7130):499-504.

31. Leon DA. Fetal growth and adult disease.European Journal of Clinical Nutrition. 1998 Jan;52:S72-8.

32. Lucas A. Programming by early nutrition in man. The Childhood Environment and Adult Disease. 1991 May 3;1991:38-55.

33. Rasmussen KM. The “fetal origins” hypothesis: challenges and opportunities for maternal and child nutrition. Annual Review of Nutrition. 2001 Jul;21(1):73-95.

34. Waterland RA, Garza C. Potential mechanisms of metabolic imprinting that lead to chronic disease. The American Journal of Clinical Nutrition. 1999 Feb 1;69(2):179-197.

35. Waterland RA, Jirtle RL. Early nutrition, epigenetic changes at transposons and imprinted genes, and enhanced susceptibility to adult chronic diseases.Nutrition. 2004 Jan 1;20(1):63-8.

36. Jenkins S, Horner SD. Barriers that influence eating behaviors in adolescents. Journal of Pediatric Nursing. 2005 Aug 31;20(4):258-267.

37. Scal P. Transition for youth with chronic conditions: primary care physicians’ approaches. Pediatrics. 2002 Dec 1;110(Supplement 3):1315-21.

38. Dalstra JA, Kunst AE, Borrell C, Breeze E, Cambois E, Costa G, et al. Socioeconomic differences in the prevalence of common chronic diseases: an overview of eight European countries. International Journal of Epidemiology. 2005 Apr 1;34(2):316-326.

39. Cavill I, Auerbach M, Bailie GR, Barrett-Lee P, Beguin Y, Kaltwasser P, Littlewood T, Macdougall IC, Wilson K. Iron and the anaemia of chronic disease: a review and strategic recommendations. Current Medical Research and Opinion. 2006 Apr 1;22(4):731-7.

40. Roy CN. Anemia of inflammation.ASH Education Program Book. 2010 Dec 4;2010(1):276-280.

41. Mockenhaupt FP, Rong B, Günther M, Beck S, Till H, Kohne E, Thompson WN, Bienzle U. Anaemia in pregnant Ghanaian women: importance of malaria, iron deficiency, and haemoglobinopathies. Transactions of the Royal Society of Tropical Medicine and Hygiene. 2000 Sep 1;94(5):477-483.

42. Bentley ME, Griffiths PL. The burden of anemia among women in India.European Journal of Clinical Nutrition. 2003 Jan 1;57(1):52-60.

43. Story M, Kaphingst KM, Robinson-O'Brien R, Glanz K. Creating healthy food and eating environments: policy and environmental approaches. Annu.Rev. Public Health. 2008 Apr 21;29:253-272.

44. Hawkes C. Uneven dietary development: linking the policies and processes of globalization with the nutrition transition, obesity and diet-related chronic diseases. Globalization and Health. 2006 Mar 28;2(1):4.

45. Cavill N, Biddle S, Sallis JF. Health enhancing physical activity for young people: Statement of the United Kingdom Expert Consensus Conference. Pediatric Exercise Science. 2001 Feb;13(1):12-25.

46. Booth FW, Chakravarthy MV, Gordon SE, Spangenburg EE. Waging war on physical inactivity: using modern molecular ammunition against an ancient enemy. Journal of Applied Physiology. 2002 Jul 1;93(1):3-30.

47. Chakravarthy MV and Booth FW. Exercise. Philadelphia, PA: Elsevier, 2003.

48. Bouchard C, Blair SN, Haskell WL, Lee IM. Dose-response Issues Concerning Physical Activity & Health. Medicine & Science in Sports & Exercise. 2001 May 1;33(5):S226.

49. Loucaides CA, Chedzoy SM, Bennett N. Differences in physical activity levels between urban and rural school adolescents in Cyprus. Health Education Research. 2004 Apr 1;19(2):138-47.

50. Warren CW, Jones NR, Eriksen MP, Asma S, Global Tobacco Surveillance System (GTSS) collaborative group. Patterns of global tobacco use in young people and implications for future chronic disease burden in adults. The Lancet. 2006 Mar 10;367(9512):749-753.

51. Viswanathan M, Golin CE, Jones CD, Ashok M, Blalock SJ, Wines RC, Coker-Schwimmer EJ, Rosen DL, Sista P, Lohr KN. Interventions to improve adherence to self-administered medications for chronic diseases in the United States: a systematic review. Annals of Internal Medicine. 2012 Dec 4;157(11):785-95.

52. Roberts CK, Barnard RJ. Effects of exercise and diet on chronic disease.Journal of Applied Physiology. 2005 Jan 1;98(1):3-30.

53. Yach D, McKee M, Lopez AD, Novotny T. Improving diet and physical activity: 12 lessons from controlling tobacco smoking. British Medical Journal. 2005 Apr 14;330(7496):898.

54. Brownson RC, Haire-Joshu D, Luke DA. Shaping the context of health: a review of environmental and policy approaches in the prevention of chronic diseases. Annu Rev Public Health. 2006 Apr 21;27:341-370.

55. Desroches S, Lapointe A, Ratté S, Gravel K, Légaré F, Turcotte S. Interventions to enhance adherence to dietary advice for preventing and managing chronic diseases in adults. The Cochrane Library.2013 Jan 1.

56. Radhakrishnan K. The efficacy of tailored interventions for self‐management outcomes of type 2 diabetes, hypertension or heart disease: a systematic review. Journal of advanced nursing. 2012 Mar 1;68(3):496-510.

57. Weingarten SR, Henning JM, Badamgarav E, Knight K, Hasselblad V, GanoJr A, Ofman JJ. Interventions used in disease management programmes for patients with chronic illness which ones work? Meta-analysis of published reports.British Medical Journal. 2002 Oct 26;325(7370):925.

58. Lang A, Froelicher ES. Management of overweight and obesity in adults: behavioral intervention for long-term weight loss and maintenance. European Journal of Cardiovascular Nursing. 2006 Jun;5(2):102-14.

59. Harris KJ, Paine-Andrews A, Richter KP, Lewis RK, Johnston JA, James V, Henke L, Fawcett SB. Reducing elementary school adolescents risks for chronic diseases through school lunch modifications, nutrition education, and physical activity interventions. Journal of Nutrition Education. 1997 Jul 1;29(4):196-202.

60. Prabhushankar T, Thakur JS, Jaswal N, Bharti B, Bhansali A. Effect of 12-Week Lifestyle Intervention on Behavioral, Anthropometry and Biochemical Profile of School Adolescents in Chandigarh. India. J Community Med Health Educ. 2015;5(367):2161-0711.

61. Beratarrechea A, Lee AG, Willner JM, Jahangir E, Ciapponi A, Rubinstein A. The impact of mobile health interventions on chronic disease outcomes in developing countries: a systematic review. Telemedicine and e-Health. 2014 Jan 1;20(1):75-82.

62. Puska P, Vartiainen E, Pallonen U, Salonen JT, Pöyhiä P, Koskela K, McAlister A. The North Karelia youth project: evaluation of two years of intervention on health behavior and CVD risk factors among 13-to 15-year old adolescents . Preventive Medicine. 1982 Sep 1;11(5):550-570.

63. Thakur JS, Bharti B, Tripathy JP, Dhawan V, Bhansali A. Impact of 20 Week Lifestyle Intervention Package on Anthropometric Biochemical and Behavioral Characteristics of Schoolchildren in North India. Journal of tropical pediatrics. 2016 Apr 22;62(5):368-76.

64. Daivadanam M, Wahlstrom R, Ravindran TS, Sarma PS, Sivasankaran S, Thankappan KR. Design and methodology of a community-based cluster-randomized controlled trial for dietary behaviour change in rural Kerala. Global health action. 2013 Dec 1;6(1):20993.

65. LaMonte MJ, Blair SN, Church TS. Physical activity and diabetes prevention. Journal of Applied Physiology. 2005 Sep 1;99(3):1205-13.

66. Sallis J, Bauman A, Pratt M. Environmental and policy interventions to promote physical activity. American Journal of Preventive Medicine. 1998 Nov 30;15(4):379-397.

67. Schmid TL, Pratt M, Howze E. Policy as intervention: environmental and policy approaches to the prevention of cardiovascular disease. American Journal of Public Health. 1995 Sep;85(9):1207-11.

68. Glanz K, Lankenau B, Foerster S, Temple S, Mullis R, Schmid T. Environmental and policy approaches to cardiovascular disease prevention through nutrition: opportunities for state and local action. Health Education & Behavior. 1995 Nov 1;22(4):512-527.

69. King AC, Jeffery RW, Fridinger F, Dusenbury L, Provence S, Hedlund SA, Spangler K. Environmental and policy approaches to cardiovascular disease prevention through physical activity: issues and opportunities. Health Education Quarterly. 1995 Nov;22(4):499-511.

70. Wechsler H, Devereaux RS, Davis M, Collins J. Using the school environment to promote physical activity and healthy eating. Preventive Medicine. 2000 Aug 1;31(2):S121-137.

71. Harrell JS, McMurray RG, Bangdiwala SI, Frauman AC, Gansky SA, Bradley CB. Effects of a school-based intervention to reduce cardiovascular disease risk factors in elementary-school adolescents : the Cardiovascular Health in Adolescents (CHIC) study. The Journal of Pediatrics. 1996 Jun 30;128(6):797-805.

72. Gaziano TA, Galea G, Reddy KS. Scaling up interventions for chronic disease prevention: the evidence.The Lancet. 2007 Dec 14;370(9603):1939-46.

73. Brownson RC, Haire-Joshu D, Luke DA. Shaping the context of health: a review of environmental and policy approaches in the prevention of chronic diseases. Annu Rev Public Health. 2006 Apr 21;27:341-70.

74. Ramachandran A, Snehalatha C. Current scenario of diabetes in India. Journal of Diabetes. 2009 Mar 1;1(1):18-28.

75. Camaschella C. Iron-deficiency anemia. N Engl J Med. 2015; 372: 1832–1843.

76. SerjeantGR,Serjeant BE. Sickle Cell Disease; Oxford University Press: Oxford, UK; New York, NY, USA, 1992.

77. Kassebaum NJ, Jasrasaria R, Naghavi M, Wulf SK, Johns N, Lozano R, Regan M, Weatherall D, Chou DP, Eisele TP, Flaxman SR. A systematic analysis of global anemia burden from 1990 to 2010.Blood. 2014 Jan 30;123(5):615-624.

78. Machado AP, Lima BM, Laureano MG, Silva PH, Tardin GP, Reis PS, Santos JS, JácomoNeto D, D'Artibale EF. Educational strategies for the prevention of diabetes, hypertension, and obesity.Revista da AssociaçãoMédicaBrasileira. 2016 Nov;62(8):800-8.

79. Berenson GS, Srinivasan SR, and Webber LS. Prognostic significance of lipid profiles in adolescents , In: “Childhood Prevention of Atherosclerosis and Hypertension” Latter RM and Shekelle RB, Eds. Raven Press, New York, 1980.

80. Lauer RM, and Clarke WR. Immediate and long-term prognostic significance of childhoodblood pressure levels, In: Childhood Prevention of Atherosclerosis and Hypertension Lauer RM and Shekelle RB, Eds. Raven Press, New York, 1980.

81. Gupta R. Trends in hypertension epidemiology in India. Journal of Human Hypertension. 2004 Feb 1;18(2):73-78.

82. Peltzer K, Pengpid S. Fruits and vegetables consumption and associated factors among in-school adolescents in five Southeast Asian countries. Int J Environ Res Public Health. 2012 Oct 11;9(10):3575-87. doi: 10.3390/ijerph9103575.

83. Kumar P, Kumar D, Ranjan A, Singh CM, Pandey S, Agarwal N. Prevalence of Hypertension and its Risk Factors Among School Going Adolescents of Patna, India. J Clin Diagn Res. 2017 Jan;11(1):SC01-SC04. doi: 10.7860/JCDR/2017/23886.9196. Epub 2017 Jan 1.

84. Ha K, Chung S, Lee HS, Kim CI, Joung H, Paik HY, Song Y. Association of Dietary Sugars and Sugar-Sweetened Beverage Intake with Obesity in Korean Children and Adolescents. Nutrients. 2016 Jan 8;8(1):31. doi: 10.3390/nu8010031.

85. Global School-Based Student Health Survey, India (CBSE) 2007 (Age 13-15)

86. National Survey on Drug Use and Health (NSDUH), 2013, (Age 12 or older for Asian population)

87. Conceptualization of the precede-proceed model of health| Open-i [Internet]. Openi.nlm.nih.gov. 2017 [cited 31 March 2017]. Available from: https://openi.nlm.nih.gov/detailedresult.php?img=PMC2686324_cia-2-677f1&req=4.

**PARENTAL INFORMED ASSENT FORM**

**Project title:** Health Promotion Intervention Package for Prevention of Behavioral Risk Factors of Chronic Diseases: A Cluster Randomized Controlled Trial among Adolescents in School Settings

**Introduction**
Your child and you have been invited to join a research study entitled, ‘Health Promotion Intervention Package for Prevention of Behavioral Risk Factors of Chronic Diseases: A Cluster Randomized Controlled Trial among Adolescents in School Settings’ by Ms. Sandeep Kaur, PhD Scholar, School of Public Health, PGIMER, Chandigarh. Please take whatever time you need to discuss the study with your family and friends, or anyone else you wish to. The decision to let you and your child join, or not to join is up to you. In this research study, we are providing health promotion intervention to promote healthy behavioral practices among school going children, their parents & teachers to prevent the risk factors of various chronic diseases

**What is involved in the study?**

Your child and you will be asked to answer some health behavior related questions pertaining to dietary intake, physical activity, tobacco & alcohol use, etc. His/her and your physical and anthropometric measurements will also be taken. We think this will take him/her and you around 30-45 minutes each. Your child and yours blood pressure will be measured. Your child can stop participating at any time.  If your child stops, he/she will not lose any benefits.

**Risks**
This study does not involve any physical and psychological risks.

**Benefits of being a part of this study?**

It is reasonable to expect the following benefits from this research: Benefit is that you and your child will get knowledge and learn the ways in which you can maintain your health and improve it by adapting to healthier behavioral practices and hence reducing the risk of getting these behavioral diseases in future and their maintenance, if already suffering from any.

Others may benefit in the future from the information we find in this study.

**Confidentiality**
your child’s and your name will not be used when data from this study are published but a code (a number) would be used purely for the purpose of monitoring the data collection process and will not be revealed beyond the study team.  Every effort will be made to keep clinical records, research records, and other personal information confidential.

**Your child rights as a research participant?**

Participation in this study is voluntary. Your child has the right not to participate at all or to leave the study at any time. Deciding not to participate or choosing to leave the study will not result in any penalty or loss of benefits to which your child is entitled, and it will not harm his/her relationship with his/ her school authorities.

**Permission for a Child to Participate in Research**

As parent or legal guardian, I authorize _________________________________ (child’s name) to become a participant in the research study described in this form.

Child’s Date of Birth

Parent or Legal Guardian’s Signature                        Date

**CONSENT FORM**

**Project title:** Health Promotion Intervention Package for Prevention of Behavioral Risk Factors of Chronic Diseases: A Cluster Randomized Controlled Trial among Adolescents in School Settings

**Principal Investigator:** Ms. Sandeep Kaur

We are doing a research study to promote healthy behavioral practices among school going children, their parents & teachers to prevent risk factors of various chronic diseases. If you decide that you want to be part of this study, you will be asked to answer some health behavior related questions pertaining to dietary intake, physical activity, alcohol & tobacco use, etc . Your physical and anthropometric measurements will also be taken. We think this will take you around 30-45 minutes. Your blood pressure will be measured.

No physical as well as psychological risks are involved in the present study.

Everyone who takes part in this study will benefit.  A benefit means that something good happens to you.  We think that the benefit for you will be that you will be get knowledge and learn the ways in which you can maintain your health and improve it by adapting to healthier behavioral practices and hence reducing the risk of getting these lifestyle diseases in future and in their maintenance, if already suffering from any.

When we are finished with this study, we will write a report about what was learned. This report will not include your name or that you were in the study.

You do not have to be in this study if you do not want to be.  You can stop participating at any time of the study. Your parents know about the study too.

If you decide you want to be in this study, please sign your name.

I, _________________________________, want to be in this research study.

___________________________________          _________
               (Sign your name here)                                   (Date)
