# Supplementary material for: Health promotion intervention to prevent risk factors of chronic diseases: Protocol for a cluster randomized controlled trial among adolescents in school settings of Chandigarh (India)
Source: PLoS One. 2022 Feb 17;17(2):e0263584. doi: 10.1371/journal.pone.0263584 (PMC8853575; doi:10.1371/journal.pone.0263584)
Supplement: Attachment — Submitted filename: Response to Reviewer.docx [file pone.0263584.s004.docx]

**RESPONSE TO THE REVIEWER’S COMMENTS**

**Response to The Academic Editor’s Comments**

We thank the editor for the comments and for coordinating the review process. We have revised the manuscript and addressed the comments raised by the academic editor. The response to each of the comments is provided in blue colour. The changes have been made in the manuscript using the ‘track changes’ command.

1. *Low-middle income is the best definition for in developing countries*

We agree with the editor’s comment and have changed the term developing countries with a more appropriate term, the ‘low- and middle-income’ countries

2. *About the statement that there are almost no studies from middle-income countries: There are few studies conducted in Brazil as shown in the pooled analysis
   School-based obesity interventions in the metropolitan area of Rio De Janeiro, Brazil: pooled analysis from five randomised studies.*

*Rodrigues RDRM, Hassan BK, Sgambato MR, Souza BDSN, Cunha DB, Pereira RA, Yokoo EM, Sichieri R.Br J Nutr. 2021 Nov 14;126(9):1373-1379. doi: 10.1017/S0007114521000076. Epub 2021 Jan 14.PMID: 33441203.*

Thank you for bringing to our notice this recent publication (2021). We have added it in line 84 and cited the study with reference number 13.

3. *I think it is still hard to understand the cluster allocation to treatment or control “The average income of the clusters will be used to categorise them into different income quartiles. Then clusters having similar socio-economic status would be grouped in blocks. Lastly, half of the clusters from each block will be randomly allocated to the intervention arm and half to the control arm so that the intervention and control cluster caters to a similar socio-economic group”. Only 12 schools will be selected. At best it will be 2 schools per quintile. This is a paired intervention with necessary complex analysis. I would think that choosing a more homogenous population of schools and randomly allocating them to control or intervention would be a more adequate approach.*

We thank the editor for providing us with a chance to clarify this important aspect of sampling design.

There are 115 public schools in the Union Territory of Chandigarh. Data regarding socio-economic variables of students’ households in these 115 schools are not available for the selection of homogenous clusters. Primary data collection on these variables in all 115 public schools for selecting homogenous clusters will be expensive and time-consuming.

As the number of clusters to be randomised in both arms are small, i.e., 12 clusters, in random selection, the distribution of socio-economic variables among the clusters selected in the two arms of the study may not be similar, which may confound the trial’s outcome. Hence, in view of limited resources available for the study, we would collect data only in the randomly selected clusters of 12 schools out of the total 115 schools. The data on households economic status will be used to stratify the clusters. Then, using the stratified random sampling method, clusters will be chosen to have a similar distribution of confounding variables in intervention and control arm clusters. We agree that this is a complex sampling design, but in view of the limited resources available, we think the proposed sampling design is more suitable for our study.

We have revised the manuscript to convey the sampling design and analysis in a better manner (lines 129 to 133 and 464) and have added a reference (no.24) in the manuscript that supports the use of stratified random sampling in cRCTs specially when the sample size is small.
